# Factors influencing adherence to the new intermittent preventive treatment of malaria in pregnancy policy in Keta District of the Volta region, Ghana

**DOI:** 10.1186/s12884-019-2544-8

**Published:** 2019-11-20

**Authors:** Alren O. Vandy, Nana Yaw Peprah, Joseph Yaw Jerela, Perfect Titiati, Abubakar Manu, Joseph Akamah, Ernest T. Maya, Kwasi Torpey

**Affiliations:** 1 University of Ghana College of Health Sciences, Accra, Ghana; 2 National Malaria Control Programme, Ghana Health Service, Accra, Ghana; 3 Keta Municipal Health Directorate, Keta, Ghana

**Keywords:** Malaria, Adherence, IPTp-SP, Volta region, Ghana

## Abstract

**Background:**

About 25% of pregnant women in malaria-endemic areas are infected with malaria and this accounts for about 15% of maternal deaths globally. Intermittent preventive treatment in pregnancy with sulfadoxine-pyrimethamine (IPTp-SP) is one of the main strategies for prevention of malaria in pregnancy. A new recommendation was made by the World Health Organization (WHO) that at least three doses of IPTp-SP should be administered before delivery. This study sought to determine the factors influencing adherence to the new IPTp-SP policy in Keta District, Volta region, Ghana.

**Methods:**

A cross-sectional quantitative study among 375 nursing mothers at four selected health facilities in Keta district, Ghana was conducted using a structured questionnaire to interview participants. Sampling proportionate to the size of facility was used to determine the number of nursing mothers from each facility based on the caseload. For each facility systematic random sampling was used to select eligible nursing mothers. Data was analyzed using STATA 15. Chi-square was used to test bivariate association between categorical variables and adherence. Logistic regression analysis was used to examine sociodemographic, individual and institutional factors influencing adherence to IPTp-SP.

**Result:**

About 82.1% of participants adhered to the WHO policy recommendations of at least three doses of IPTp-SP. However, only 17.1% received Ghana’s five dose coverage recommendation. The proportion of IPTp-SP coverage for IPTp1 was 98.9%; IPTp2 95.5%; IPTp3 80.8%; IPTp4 39.5%; IPTp5 17.1%.

**Conclusion:**

Adherence to IPTp-SP was satisfactory according to WHO’s policy recommendation, however, majority of the participants had less than the five doses recommended in Ghana. Number of Antenatal Care (ANC) visits and knowledge of malaria were the main determinants of adherence to IPTp-SP.

## Background

Malaria is a life-threatening disease that is caused by a parasitic protozoan, plasmodium. It is endemic in 91 countries and nearly 50% of the world’s population at the start of 2016 were susceptible to malaria [1]. Majority of malaria reported deaths occurs in sub-Saharan Africa and about 212 million new cases and 429,000 deaths were reported globally [2].

In Sub Saharan Africa with at least 25% of pregnant women infected with malaria in areas endemic for malaria and it accounts for 15% of maternal deaths globally [2].

About 25 million women in Sub Saharan Africa become pregnant each year and are at risk of malaria infection [3]. Ten thousand of these pregnant women and 200,000 of newborns die due to malaria in pregnancy [4].

Vulnerability of pregnant women to malaria is associated with hormonal and immunological changes in pregnancy [5]. First and second pregnancies are more susceptible to malaria and the level of parasitaemia decreases with increasing numbers of pregnancies [6].

Studies in Ghana have shown that malaria during pregnancy increases maternal anemia and low birth weight especially among women living in rural communities [7]. It is a major cause of morbidity and mortality with an estimated 382,862 pregnant women suffering from malaria in 2016 [8]. The Volta region in Ghana has been known to be one of the highest malaria prevalence regions in Ghana [9]. In 2017, the Volta region recorded the lowest score for 2016 Ghana Health Service National Health League Table and performed poorly in maternal health service delivery [10].

WHO recommends three main strategies for the treatment and prevention of malaria during pregnancy. These approaches are: intermittent preventive treatment in pregnancy (IPTp) with sulfadoxine-pyrimethamine (SP), using long lasting insecticidal nets (LLINs), and early diagnosis and treatment of malaria cases [11].

Prevention of malaria in pregnancy forestalls complications of severe malaria and reduces the risk to both pregnant women and their unborn child.

Chemoprophylaxis with IPTp-SP improves the maternal and neonatal outcomes and is recommended in regions where there is moderate to high transmission of malaria [12].

In 2007, the first WHO recommendation policy on IPTp-SP was made and it recommended that all pregnant women attending antenatal care (ANC) services should be given two doses of SP during pregnancy. The first dose of SP should be given at the start of the second trimester and second dose at the start of the third trimester. It should be given as directly observed treatment (DOT) during antenatal visits [12]. In Ghana, three doses of SP was given to pregnant women from 16 to 36 weeks [13]. In 2012, WHO Evidence Review Group reviewed new evidence from published and unpublished studies on IPTp with SP and agreed that more than two doses would be more effective in preventing malaria in pregnancy. A new recommendation was made that the first dose of IPTp-SP should be administered as early as possible in the second trimester and each dose of SP should be given at least 1 month apart up until delivery [14]. This update was done to maximize the number of SP doses given. However, according to WHO, about 69% of pregnant women in sub-Saharan Africa did not have access to the recommended three or more doses of IPTp-SP [11].

Ghana transitioned from the three doses to the five dose IPTp-SP recommendation in 2014 [15]. Currently, only a few studies have focused on the implementation of the new IPTp-SP policy that was adopted.

This study sought to determine the factors influencing adherence to the new IPTp-SP policy in Keta District, Volta region, Ghana.

## Methods

### Study design, population and setting

A facility-based cross-sectional quantitative study was conducted in May–June, 2018 to examine the factors influencing adherence to the new IPTp-SP policy. Participants were nursing mothers who had delivered within 3 months and attending the Child Welfare Clinics (CWC) and Postnatal Clinic at four health facilities in the Keta District of the Volta region, Ghana.

### Sample size calculation and sampling procedures

A minimum sample size of 360 was computed with the Cochran (1977) formula, using an IPTp3 prevalence of 37.5% in the Volta region (NMCP 2017); 95% confidence interval; a margin of error of 5%.

Four health facilities with the highest volume of antenatal attendees and nursing mothers were purposively selected as recruitment sites out of twenty-eight facilities.

A sampling proportional to the number of nursing mothers seen per facility was used to determine the number of respondents recruited from each of the four facilities. Nursing mothers who gave written consent and had delivered within 3 months prior to data collection and visiting postnatal clinic or CWC at each selected health facilities were interviewed. Systematic random sampling was used for the selection of nursing mothers at the CWC who met the criteria. The sampling interval was determined by using estimated average clinic attendance per month and facility sample size. The first client was randomly selected by balloting and the interval applied for nursing mother attending the CWC until the sample size was reached.

### Data collection

An interviewer-administered questionnaire was used to collect information on socio-demographic characteristics of the nursing mothers, knowledge on malaria in pregnancy, knowledge on IPTp and SP doses (Additional file 1). Gestational age at first ANC visit, number of ANC visits and number of SP doses before delivery was extracted from the ANC Card. Information on health system/institutional factors were also elicited using a questionnaire administered to staff of antenatal clinic (Additional file 2). Table 1 shows the list of study variables, indicators, operational definition and scale of measurement.

**Table 1:** List of Study Variables

Variables	Indicators	Operational definition	Scale of Measurement
Adherence to IPTp	Adherence	3 or more doses of IPTp-SP	Binary
Sociodemographic	Age	Age of respondents in years	Continuous
	Marital Status	Marital status of respondents	Categorical
	Religion	Religion of respondents	Categorical
	Educational Level	Educational level attained	Categorical
	Employment status	Unemployed, Employed or Self-employed	Categorical
	Occupation	Current occupation of respondents	Categorical
	Parity	Number of live births	Continuous
ANC Attendance and Obstetric Characteristics	Number of ANC Visits	Number of ANC visits during most recent pregnancy	Binary
	Gestational age at first ANC visit	The age of pregnancy (fetus) in weeks at which first ANC visit was made	Continuous
	Gestational age at first IPTp-SP dose	The age of pregnancy (fetus) in weeks at which first IPTp-SP dose was received	Continuous
Individual factors	Knowledge level on Malaria, MiP and IPTp	Knowledge measured by answers to questions	Categorical
Health System / Institutional factors	SP availability	Availability of SP at time of data collection	Binary
	Access to IPTp Services	Provision of IPTp-SP services at health facility	Binary
	Knowledge level of ANC Health Workers on new IPTp-SP policy recommendation	Knowledge measured by answers to questions.	Categorical
	Training of ANC Healthcare Workers	ANC Healthcare workers who had formal training on IPTp.	Binary
	DOT practices	Practice of DOT for IPTp delivery.	Binary

## Measures

### Outcome variables

Primary outcome measure is the adherence to the new IPTp-SP policy in the district.

Secondary outcome measures include knowledge level of IPTp-Sp policy among pregnant women.

### Data management and analysis

Data were entered and cleaned in Microsoft Excel 2016, and exported to Stata 15 for analysis.

Descriptive statistics using frequencies and percentage were used to describe the background characteristics the respondents. Variables were considered for inclusion in the multiple logistic regression model if their *p*-values were 0.05 in the bivariate analyses to control for the confounding and to determine factors independently associated with adherence to IPTp-SP. Adjusted odd ratios (AOR) and their 95% confidence intervals were used to assess the strength of association. In all analyses, a *p*-value of 0.05 was used to determine statistical significance. Knowledge of malaria in pregnancy was measured by asking questions about malaria transmission and interventions with a total score of 13. Scores of 0–5 was considered poor, 5–10 fair and more than 10 was good. All analyses were performed in Stata 15 (StataCorp LLC, College Station, Texas).

## Results

### Socio-demographic characteristics of study participants

A total of 375 nursing mothers within the reproductive age group (15–49 years) in the 4 selected health facilities were interviewed for the study. Table 2 summarizes the socio-demographic characteristics of the participants.

**Table 2:** Sociodemographic characteristics of participants

Variable	Frequency (*n*)	Percentage (%)
Age grouping		
<18	9	2.4
18–25 years	132	35.2
26–35 years	199	53.1
36–45 years	35	9.3
Total	375	100
Marital Status		
Single	24	6.4
Married	344	91.7
Divorce / Separated	3	0.8
Widowed	4	1.1
Total	375	100
Educational level		
No formal education	61	16.3
Primary	119	31.7
Secondary	169	45.1
Bachelor’s degree	18	4.8
Post graduate degree	2	0.5
Others	6	1.6
Total	375	100
Employment Status		
Employed	40	10.7
Self-employed	236	62.9
Unemployed	99	26.4
Total	375	100
Occupation		
Trader	111	40.1
Teacher	22	7.9
Artisan	94	33.9
Farmers / Fishmongers	23	8.3
Healthcare Workers	5	1.8
Others	22	7.9
Total	277	100
Religion		
Christians	331	88.3
Muslims	14	3.7
Traditionalist	30	8.0
Total	375	100
Parity		
1	101	26.9
2	118	31.5
≥3	155	41.3
Missing data	1	0.3
Total	375	100

In all, 53.1% (199/375) of the participants were between the ages of 26–35 years and the majority (91.7%, 344/375) were married with 41.3% (15/375) having three or more children. Nearly half of them had secondary level education 45.1%, (169/375) and most of the participants (62.9%, 236/375) were self-employed as traders and artisans.

Majority of the participants 88.1% (331/375) were Christians followed by traditionalist (8.0%, 30/375) and then Muslims (3.7%,14/375) (Table 2).

### ANC attendance and obstetric characteristics of participants

Table 3 presents descriptive information on ANC attendance and obstetrics characteristics of the nursing mothers. About two-thirds of the nursing mothers (66.1%, 248/375) had less than the eight WHO recommended ANC visits/contacts during pregnancy, 33.9% (127/375) had eight or more ANC visits during the period of pregnancy. The mean number of ANC was 6.5 ± 2.6 visits.

**Table 3:** ANC and Obstetric Characteristics of participants

Variable	Frequency(*n*)	Percentage (%)
Gestational age at first ANC		
≤13 weeks	165	44.0
13–26 weeks	199	53.1
≥26 weeks	11	2.9
Total	375	100
Number of ANC visits		
<8	248	66.1
≥8	127	33.9
Total	375	100
Gestational age at first IPTp-SP		
≤13 weeks	11	3.0
13–26 weeks	339	90.4
≥26 weeks	21	5.6
Don’t know	4	1.1
Total	375	100
Number of IPTp-SP doses		
0	4	1.1
1	13	3.5
2	50	13.3
3	160	42.7
4	84	22.4
≥5	64	17.1
Total	375	100
Number of SP tablet given per dose		
≤2	29	7.7
3	330	88.0
>3	12	3.2
Don’t know	4	1.1
Total	375	100

*n* number of respondents, *ANC* antenatal care, *IPTp-SP* Intermittent Preventive Treatment in Pregnancy with sulphadoxine pyrimethamine, *SP*- sulphadoxine pyrimethamine

More than half (53.1%, 199/375) of the participants had their first ANC visit during the second trimester (13–26 weeks), 44.0% (165/375) during the first trimester and only 2.9% (11/375) had their first ANC visit in the third trimester. The mean gestational age at first ANC visit was 14.4 ± 6.5 weeks.

In all, 90.4% (339/375) of the respondents had their first dose of IPTp-Sp during the second trimester between 13 and 26 weeks of gestation and 21 (5.7%) respondents had theirs in the third trimester of pregnancy.

Thirteen respondents (3.5%; 13/375) had one dose of IPTp-SP with the majority (42.7%, 160/375) having three doses of IPTp-SP before delivery. About 17.1% (64/375) had five doses or more of IPTp-SP before delivery. Only four respondents (1.1%) did not take SP during their pregnancy. Some respondents reported side effects and allergies after taking SP tablets.

When asked the number of SP tablets taken per dose, (88.0%, 330/375) reported of receiving three tablets of SP per dose, 29 (7.7%) reported of receiving less than three SP tablets and 12 (3.2%) mentioned more than three SP tablets per dose (Table 3).

### Adherence to new IPTp-SP policy recommendations and proportion of IPTp-SP coverage

Majority of the participants (82.1%, 308/375) had three or more doses of IPTp-SP during their pregnancy as recommended by WHO and only 17.9% (67/375) had less than three doses.

However, when using Ghana’s five dose IPTp-SP coverage recommendation, only 64 (17.1%) participants adhered.

**Fig. 1:**
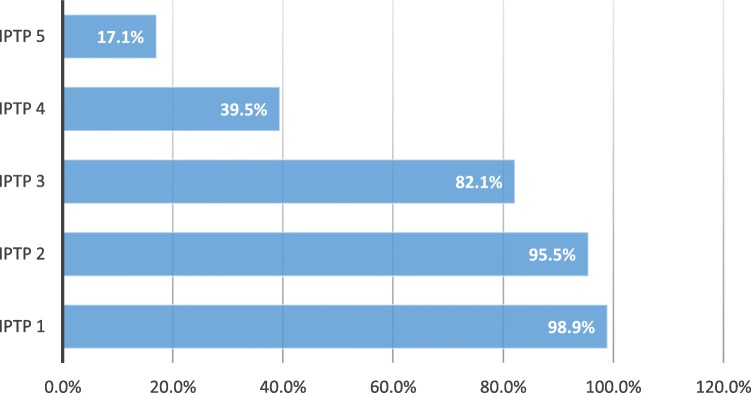
Coverage of IPTp-SP

The proportion of IPTp-SP coverage for IPTp1 was 98.9%; IPTp2 95.5%; IPTp3 82.1% IPTp4 39.5%; IPTp5 17.1% (Fig. 1).

### Individual factors

Over half of the respondents (52.0%) had a fair knowledge about malaria and Malaria in Pregnancy (MiP), 42.8% had poor knowledge and only 5.2% had good knowledge about malaria and MiP. Majority (83.5%, 308/375) of respondents had knowledge about the IPTp-SP and the benefits of it. The main source of information on malaria was from ANC/Health facility followed by the media.

### Healthcare system/institutional factors

All the selected health facilities offered IPTp services (both static and outreach services).

SP was the drug of choice at these facilities; however, one out of the four facilities did not have SP at the time the study was conducted but was available few months prior to data collection.

SP was given as DOT at all the health facilities where the study was conducted and clean drinking water (sachets) was available for pregnant women. Of the 11 staff interviewed, six (54.6%) of respondents have had training on IPT-SP.

Assessment of Knowledge of ANC staff on IPTp showed that 90.9% of the ANC workers knew the correct definition of IPTp. All respondents (100.0%) knew the recommended drug, the dose and the correct interval for IPTp. However, only 45.5% knew when to start IPTp and 81.8% knew when it was contraindicated during pregnancy. This is shown in Table 4.

**Table 4:** Knowledge of ANC Workers on IPTp-SP

Knowledge *N* = 11	Frequency	Percentage (%)
Correct definition of IPTp	10	90.9
Recommended drug for IPTp in Ghana	11	100.0
When to start IPTp	5	45.5
When not to give IPTp	9	81.8
Recommended dose for IPTp in Ghana	11	100.0
Correct interval for IPTp	11	100.0

### Factors associated with adherence to IPTp-SP

In the multivariate logistic regression model, after adjusting for characteristics of participants, having ≥8 ANC visits (AOR = 4.51, 95% CI 1.76–11.57, *p* < 0.05) and knowledge of IPTp-SP (AOR = 2.74, 95% CI 1.29–5.82, *p* < 0.05) were significantly associated to adherence to IPTp-SP. This is shown in Table 5.

**Table 5:** Multivariate analysis of factors associated with adherence

	AOR	95% CI	p
Age grouping			
*<18*	Ref		
18–25	2.46	0.43–14.13	0.311
26–35	5.50	0.94–32.08	0.058
36–45	4.49	0.58–34.81	0.151
Religion			
*Christian*	Ref		
Muslim	1.00		
Traditionalist	0.52	0.19–1.42	0.201
Educational level			
*No formal education*	Ref		
Primary	0.86	0.36–2.05	0.734
Secondary and above	2.21	0.84–5.84	0.109
Gestational age at first ANC			
*≤13 weeks*	Ref		
13–26 weeks	1.22	0.60–2.49	0.589
>26 weeks	0.68	0.09–4.93	0.705
Gestational age at first IPTp-SP			
*≤13 weeks*	Ref		
13–26	0.94	0.17–5.35	0.946
>26	0.15	0.02–1.37	0.093
Number of ANC visits			
<8	Ref		
>=8	4.51	1.76–11.57	0.002*
Knowledge on Malaria			
*Poor*	Ref		
Average	1.34	0.67–2.67	0.402
Good	3.26	0.30–35.21	0.33
Knowledge of IPTp-SP			
*No knowledge*	Ref		
Knowledge	2.74	1.29–5.82	0.009

*AOR* adjusted odds ratio, 95% CI 95% confidence interval, *Ref* reference

*- *p* < 0.05

## Discussion

According to National Malaria Control Programme, IPTp-SP coverage for Volta region in 2017 was IPTp 1–63.7%, IPTp 2–53.0%, IPTp 3–37.5%, IPTp 4–18.8%, IPT 5–5.9% which appears to be lower than those found in the present study [16]. However, in the same year, a study conducted in the Accra Metropolitan area in Ghana had similar findings to the current study and reported IPTp1 to be 98.8%, IPTp 2–94.9%, IPTp3–87.5%, IPTp 4–55.7%, IPTp 5–14.5% [17].

In a study by Sikambale, Halwindi, & Baboo (2013) in Zambia, IPTp3 coverage was found to be much lower (30%). About a third (28.8%) had no IPTp-SP dose taken during their most recent pregnancy [18]. In our study, only 1.1% of the respondents did not have any dose of IPTp-SP and IPTp 3 coverage was much higher.

Several studies have shown an increase in adherence to IPTp-SP due to ongoing campaigns and increase in coverage. According to WHO (2016), there is an increase in adherence to the IPTp-SP policy in malaria-endemic regions and at least 50% of women reported to have received one or more doses [2]. Also, only 19% of eligible pregnant women had three or more doses of IPTp-SP compared to 18% in the previous year [2].

A study conducted in Mali on uptake of IPTp-SP found a higher uptake of SP of three doses more than the Demographic and Health Survey reported data [19]. Nonetheless, Mpogoro et al. (2014) in Tanzania identified a much lower proportion of pregnant women adhering to IPTp-SP compared to the national survey report [20].

The number of ANC visits was the main determinant of adherence to IPTp-SP in the current study though gestational age at first ANC and dose was significant. Most of the previous studies focused on the antenatal care model of four ANC visits during pregnancy which also showed significant association with adherence to IPTp-SP.

ANC visits promote uptake of IPTp-SP prevents malaria in pregnancy, protects mother and the unborn baby from complications of malaria in pregnancy and improves perinatal outcome [12, 21].

Early detection of pregnancy is important for early commencement of ANC [22]. In Ghana, there is a government policy of free maternal care and most ANC services are covered by the national health insurance scheme [23]. A study in Cameroon showed that there was a significant association between amount of SP doses taken and early first ANC attendance (at an early gestation age). Pregnant women who had early first ANC attendance were more likely to receive the recommended doses of SP [24].

Various studies have been conducted to assess the relationship between knowledge and IPTp-SP use. Studies in Zambia and Nigeria have shown that knowledge level of pregnant women on IPTp-SP strongly influences their adherence to the IPTp-SP recommendations and women were 2.6 times more likely to complete IPTp-SP doses [18, 25]. The studies also support the finding from the current study that knowledge on malaria and IPTp-SP use is average among pregnant women. This can be associated with the level of education of the respondents from the study.

To reiterate, education empowers women with knowledge which in turn enables women to make informed choices and in this case, choice of ANC and IPTp-SP use.

Institutional factors such as knowledge of the health care workers and their capacity is essential to operationalize the policy direction into public health practice. While the knowledge on IPTp was generally good, most of the healthcare workers had difficulties on when to start prophylaxis. This is particularly important as any uncertainties on when to start is likely to delay the initiation of IPTp and limit the ability to deliver five doses before delivery. Enhancing staff capacity is an important element to improve uptake and early initiation of IPTp [26].

The study was well powered and provides useful insight in the implementation of the new policy in Keta District. However, it cannot be generalized to represent the entire Volta region or Ghana. In addition, there may be recall bias from participants as the interviews took place a few months after delivery.

## Conclusions

Adherence to IPTp- SP in the study was determined to be 82.1% with WHO’s recommendation of 3 or more doses of IPTp and 17.1% with Ghana’s 5 dose policy recommendation.

Number of ANC visits during pregnancy was one of the main determinants of adherence to IPTp-SP. Majority (66.1%) of the participants had less than the recommended 8 ANC visit during pregnancy.

Health care workers had a good knowledge of IPTp but were less certain on when to start the prophylaxis.

## Supplementary information

**Additional file 1.** Questionnaire for nursing mothers

**Additional file 2.** Questionnaire for staff for antenatal clinic

## Abbreviation

ANC: Antenatal Care
AOR: Adjusted Odd Ratios
CI: Confidence Interval
CWC: Child Welfare Clinics
DOT: Directly Observed Treatment
IPTp: Intermittent Preventive Treatment in Pregnancy
IPTp-SP: Intermittent Preventive Treatment in Pregnancy with Sulfadoxine-Pyrimethamine
MiP: Malaria in Pregnancy
SP: Sulfadoxine-Pyrimethamine
WHO: World Health Organization
