# Supplementary material for: Factors influencing adherence to the new intermittent preventive treatment of malaria in pregnancy policy in Keta District of the Volta region, Ghana
Source: BMC Pregnancy Childbirth. 2019 Nov 20;19:424. doi: 10.1186/s12884-019-2544-8 (PMC6868834; doi:10.1186/s12884-019-2544-8)
Supplement: Supplementary file 1 — Additional file 1. Questionnaire for nursing mothers [file 12884_2019_2544_MOESM1_ESM.docx]

## SUPPLEMENTARY FILE 2: QUESTIONNAIRE FOR NURSING MOTHERS

**Factors influencing adherence to new intermittent preventive treatment of malaria in pregnancy policy in Keta district in Volta region, Ghana.**

| Date: | Questionnaire no: |
| --- | --- |
| Name of facility | Type of facility |

**SECTION A. SOCIO-ECONOMIC AND DEMOGRAPHIC CHARACTERISTICS**

1. Age
2. Sex:

Male 🞏 Female 🞏

1. Marital status:

Single 🞏 Married 🞏 Divorce/Separated 🞏 Widowed 🞏

1. Religion:

Christian 🞏 Muslim 🞏

Others (please specify) …. ….……………….

1. Educational Level:

No formal education 🞏 Primary 🞏 Secondary 🞏

Bachelor’s degree 🞏 Post graduate degree 🞏

Others (please specify) ……………………….

1. Employment status:

Employed 🞏 Self-employed 🞏 Unemployed🞏

1. Occupation: ………………………………………….
2. Place of residence:

Urban 🞏 Rural🞏

1. How many times have you been pregnant?

…………………………………………….

1. How many successful deliveries have you had?

………………………………………………

1. Where was your most recent delivery done?

Home🞏 Public facility 🞏 Private facility🞏

**SECTION B: KNOWLEDGE OF MALARIA IN PREGNANCY**

1. Have you heard about malaria? Yes 🞏 No🞏
2. How can you get malaria? **(You can select more than one answer)**
3. through hugging
4. Bite from infected mosquito
5. Fly
6. Evil spirit
7. Don’t know
8. Others (please specify) ………………………………
9. Have you suffered from malaria before? Yes 🞏 No 🞏
10. What are the symptoms of malaria? **(You can select more than one answer)**
11. Headache
12. Fever
13. Loss of appetite
14. Vomiting
15. Others; (please specify) ………………….
16. What are the complications of malaria in pregnancy? **(You can select more than one answer)**
17. Low birth weight
18. Still birth
19. Anemia
20. Death
21. Others (please specify) ……………….

6.How can malaria be prevented in Pregnancy? **(You can select more than one answer)**

Eating well 🞏 Use of mosquito nets 🞏

Use of mosquito spray 🞏 IPTp-SP 🞏 Proper sanitation 🞏

Others, specify: ……………………………

1. Where do you get health information about malaria in pregnancy? **(You can select more than one answer)**

ANC Clinic 🞏 Radio 🞏 TV🞏 Newspaper 🞏

Other, specify: …………………….

**SECTION C: KNOWLEDGE ABOUT IPTP-SP**

1. Have you heard about Intermittent Preventive Treatment of Malaria in Pregnancy?

Yes🞏 No 🞏

If yes, please provide information about what IPTp is …………………………………………….

1. What are some of the benefits of IPTp-SP? **(You can select more than one answer)**
2. For treatment of malaria in children
3. To prevent malaria in pregnancy
4. To treat sexually transmitted diseases
5. To prevent complication of malaria in pregnancy
6. Don’t know
7. Other, specify: …………………….
8. How many doses are required for IPTp-SP?

……………………………………………..

1. How many tablets were you given during every visit?

………………………………………….

1. Was the SP drug given under supervision?

Yes 🞏 No 🞏

1. What are some of the constraints / challenges during your ANC visit with regards to IPTp administration?

**SECTION D: RECORDS FROM ANTENATAL CARDS**

1. Gestational age at first ANC Visit……………………………………………….
2. Number of ANC visits during most recent pregnancy……………………………
3. Gestational age at which first IPTp was given ………………………………….
4. Number of IPTp-SP doses given during most recent pregnancy.

IPTp 1 🞏 IPTp 2 🞏 IPTp 3 🞏 IPTp 4🞏 IPTp 5🞏
