# Supplementary material for: Factors influencing adherence to the new intermittent preventive treatment of malaria in pregnancy policy in Keta District of the Volta region, Ghana
Source: BMC Pregnancy Childbirth. 2019 Nov 20;19:424. doi: 10.1186/s12884-019-2544-8 (PMC6868834; doi:10.1186/s12884-019-2544-8)
Supplement: Supplementary file 2 — Additional file 2. Questionnaire for staff for antenatal clinic [file 12884_2019_2544_MOESM2_ESM.docx]

## SUPPLEMENTARY FILE 3: QUESTIONNAIRE FOR ANC STAFF

**Factors influencing adherence to new intermittent preventive treatment of malaria in pregnancy policy in Keta district in Volta region, Ghana.**

| Name of facility | Sub-district |
| --- | --- |
| Type of health facility |  |

**Section A: General Information**

1. Age:
2. Sex:

Male 🞏 Female 🞏

1. Designation:

Doctor 🞏 Midwife 🞏 Nurse 🞏

Others, specify…………………………………………….

1. Number of years of practice:

Less than one year 🞏 1- 5 years 🞏 6-10 years 🞏

More than 10 years 🞏

**Section B: Knowledge of IPTp**

1. What is Intermittent Preventive Treatment of malaria in pregnancy (IPTp)?

a) Giving curative doses of an effective anti-malaria drug weekly during pregnancy

b) Giving of curative doses of an effective antimalarial drug at predefined intervals during pregnancy

c) The injection of artesunate to a pregnant woman when she has malaria

d) Giving of artesunate combined treatment (ACT) to pregnant women when they have malaria

e) Don’t know

Others, (please specify) ……………………………………………….

2. What drug is recommended for IPTp use in Ghana?

a) Chloroquine Artesunate- amodiaquine b) Fansidar (SP)

c) Lumether d) Don’t know

Others, (please specify) …………………………………………………………..

3. When is IPTp supposed to be started during pregnancy?

a) In the first trimester b) at the middle of second trimester

b) the third trimester d) At the start of the second trimester

e) Don’t know Other, (please specify) ……………………

4. At what gestation is IPTp **NOT** given during pregnancy?

a) 32 weeks b) 10 weeks

c) 36 weeks d) 38-40 weeks

e) Don’t know Others, (please specify) ……………

5. How many times during pregnancy is it recommended to give IPTp in Ghana?

a) Once b) Twice

c)Three times d) Four times

e) Five times Other, specify: …………………….

6. At what interval is it recommended that IPTp is given?

a) Monthly b) Fortnightly

c) Every three months e) Every week

Other, specify: …………………….

7. What are some of the benefits of IPTp? **(You can select more than one answer)**

a) Reduces the incidence of low birth weight infants

b) Reduces the incidence of maternal anaemia

c) Reduces the incidence of infant and maternal mortality

d) no benefit

e) others, (please specify) …………………………………………………………..

**Section 3: IPTp Services at the ANC Clinic**

1. Do you administer IPTp in your facility?

Yes 🞏 No 🞏

2. If yes, what drug is used for IPTp in your facility?

Chloroquine 🞏 Fansidar (SP) 🞏

Artesunate-amodiaquine 🞏 Lumether 🞏

3. Is the drug available at the ANC today?

Yes 🞏 No 🞏

4. How is the drug administered at your clinic?

a) Given to the pregnant women to take home

b) We observe the pregnant women take the medicine in clinic

c) Prescriptions are written for the pregnant women to go and collect at the pharmacy

d) Prescriptions are written for the pregnant women to go and buy outside the clinic

e) Other, specify .........................................................................................

5. Has there been any stock out of SP in your clinic?

Yes 🞏 No 🞏

If yes, How many times during the last year? ………………………

6. Where do you get supplies of SP for IPTp ?

a) District pharmacist b) Regional medical stores

c) Health facility in district d) Other, specify ........................................

7. Do you supply water for the pregnant women to take the drug at the ANC clinic?

Yes 🞏 No 🞏

8. If yes, what is the source of water used for pregnant women at your facility?

a) sachet water (pure water) b) bottle water

c) water from stand pipe e) water dispenser

other, (please specify) ………………………………….

9. Have you had any training in IPTp-SP before?

Yes 🞏 No 🞏

If yes, how many times in the last twelve months did you have training on IPTp-SP?

…………………………………………………

10. What are some of the challenges faced during IPTp service delivery at your facility?
